# Performance of a Computational Phenotyping Algorithm for Sarcoidosis Using Diagnostic Codes in Electronic Medical Records: Case Validation Study From 2 Veterans Affairs Medical Centers

**DOI:** 10.2196/31615

**Published:** 2022-03-02

**Authors:** Mohamed I Seedahmed, Izabella Mogilnicka, Siyang Zeng, Gang Luo, Mary A Whooley, Charles E McCulloch, Laura Koth, Mehrdad Arjomandi

**Affiliations:** 1 Division of Pulmonary, Critical Care, Allergy and Immunology, and Sleep Department of Medicine University of California San Francisco San Francisco, CA United States; 2 San Francisco Veterans Affairs Medical Center San Francisco, CA United States; 3 Department of Experimental Physiology and Pathophysiology, Laboratory of the Centre for Preclinical Research Medical University of Warsaw Warsaw Poland; 4 Department of Biomedical Informatics and Medical Education School of Medicine, University of Washington Seattle, WA United States; 5 Department of Medicine University of California San Francisco San Francisco, CA United States; 6 Measurement Science Quality Enhancement Research Initiative San Francisco Veterans Affairs Healthcare System San Francisco, CA United States; 7 Department of Epidemiology & Biostatistics University of California San Francisco San Francisco, CA United States

**Keywords:** sarcoidosis, electronic medical records, EMRs, computational phenotype, diagnostic codes, Veterans Affairs, VA, practice guidelines

## Abstract

**Background:**

Electronic medical records (EMRs) offer the promise of computationally identifying sarcoidosis cases. However, the accuracy of identifying these cases in the EMR is unknown.

**Objective:**

The aim of this study is to determine the statistical performance of using the International Classification of Diseases (ICD) diagnostic codes to identify patients with sarcoidosis in the EMR.

**Methods:**

We used the ICD diagnostic codes to identify sarcoidosis cases by searching the EMRs of the San Francisco and Palo Alto Veterans Affairs medical centers and randomly selecting 200 patients. To improve the diagnostic accuracy of the computational algorithm in cases where histopathological data are unavailable, we developed an *index of suspicion* to identify cases with a *high index of suspicion* for sarcoidosis (confirmed and probable) based on clinical and radiographic features alone using the American Thoracic Society practice guideline. Through medical record review, we determined the positive predictive value (PPV) of diagnosing sarcoidosis by two computational methods: using ICD codes alone and using ICD codes plus the *high index of suspicion*.

**Results:**

Among the 200 patients, 158 (79%) had a high index of suspicion for sarcoidosis. Of these 158 patients, 142 (89.9%) had documentation of nonnecrotizing granuloma, confirming biopsy-proven sarcoidosis. The PPV of using ICD codes alone was 79% (95% CI 78.6%-80.5%) for identifying sarcoidosis cases and 71% (95% CI 64.7%-77.3%) for identifying histopathologically confirmed sarcoidosis in the EMRs. The inclusion of the generated *high index of suspicion* to identify confirmed sarcoidosis cases increased the PPV significantly to 100% (95% CI 96.5%-100%). Histopathology documentation alone was 90% sensitive compared with *high index of suspicion*.

**Conclusions:**

ICD codes are reasonable classifiers for identifying sarcoidosis cases within EMRs with a PPV of 79%. Using a computational algorithm to capture *index of suspicion* data elements could significantly improve the case-identification accuracy.

## Introduction

### Background

Sarcoidosis is a complex disease with an unknown etiology that can involve multiple organs, and no universal or standardized measures can fully secure its final diagnosis [1-3]. In fact, it was only recently that the American Thoracic Society (ATS) published its first practice guideline to provide recommendations for diagnosing sarcoidosis and the necessary screening tests [3]. The ATS practice guideline for diagnosis requires the presence of specific clinical and radiographic features, tissue biopsy revealing nonnecrotizing granulomas, and exclusion of alternative conditions that can mimic sarcoidosis [1,3,4].

Data from electronic medical records (EMRs) are commonly used in research and by health care systems, including the United States Department of Veterans Affairs (VA), to predict outcomes or assess care quality [5]. EMR data are generally captured in two forms: (1) *structured data*, including billing codes such as the International Classification of Diseases (ICD) codes, laboratory test results, and procedural codes; and (2) *narrative or unstructured data*, including progress notes, pathology reports, and imaging reports. ICD codes cast a wider net to capture patients in the EMR because they include both inpatient and outpatient claims compared with other classifiers such as Diagnosis-Related Group that only capture inpatient claims [6]. Unstructured data contain many more details of the clinical conditions, but extracting these details is challenging and time consuming. In contrast, structured data are easier to search for, and they allow for identifying cases computationally using diagnostic codes. However, diagnostic codes can be inaccurate and difficult to verify. This is particularly true for the case definition of sarcoidosis, which is considered a diagnosis of exclusion and requires a review of clinical, radiological, and histopathological data for accurate diagnosis [3,7,8]. A few studies have reported the development of sarcoidosis-specific “computationally identifying algorithms” based on structured data elements in the EMR, although they were not validated by manual chart review [9-13]. Another study assessed the accuracy of using diagnostic codes to identify sarcoidosis cases [14] but only used the ICD, Ninth Revision (ICD-9) code and not the ICD, Tenth Revision (ICD-10) code, and it did not include any computational algorithm development. In addition, previous studies on the diagnostic accuracy of ICD codes for other common pulmonary diseases that have less or similar complexity compared with sarcoidosis, such as chronic obstructive pulmonary disease, idiopathic pulmonary fibrosis, and asthma, showed positive predictive values (PPVs) of 42%-67% [15-17]. Moreover, researchers have previously developed predictive models and risk scores to use advanced computational methods to predict, commonly, less-complex case definitions in the EMR [18-24]. For example, in a study published by Himes et al [19], Bayesian network machine learning models were constructed to predict chronic obstructive pulmonary disease. Therefore, given the complexity of securing a sarcoidosis diagnosis in the realm of real-world clinical data, it is essential to develop automated algorithms to detect confirmed and probable cases of sarcoidosis using data elements from structured and unstructured domains by incorporating the ATS diagnostic criteria [3,25].

### First Step

As the first step in evaluating the knowledge gap in developing future sarcoidosis-specific “computationally identifying algorithms,” we designed this study (1) to estimate the statistical performance of using diagnostic codes (ICD-9 and ICD-10) alone compared with a new approach that uses additional information from radiology and clinical domains, but not histopathology, to inform the utility of these codes for performing clinical phenotyping of sarcoidosis cases in large EMR data sets of the VA and (2) to assess the computational challenges in querying sarcoidosis cases and extracting high-quality sarcoidosis-related research variables from the EMR accurately.

## Methods

### Data Source and Collection

This was an observational retrospective study of EMRs available through VA Informatics and Computing Infrastructure (VINCI). VINCI provides access to comprehensive and integrated veterans’ national deidentified data sets and offers the necessary computational and analytical tools in a secure, high-performance computing environment [26,27]. This study was approved by the institutional review board of the University of California San Francisco and the Veterans Health Administration Research and Development Committee (15-16660). Patients or the public were not involved in the design, conduct, reporting, or dissemination plans of our research.

We searched the EMR data in VINCI from 1989 to 2019 and identified all patients coded as having sarcoidosis in the VA health care system, as defined by the documentation of the ICD-9 and ICD-10 codes of 135 and D86.x (including subcodes), respectively. Data were extracted through executing SQL queries in an SQL Server 2017 database. A total of 14,833 sarcoidosis cases were identified.

### Study Design

**Figure 1:**
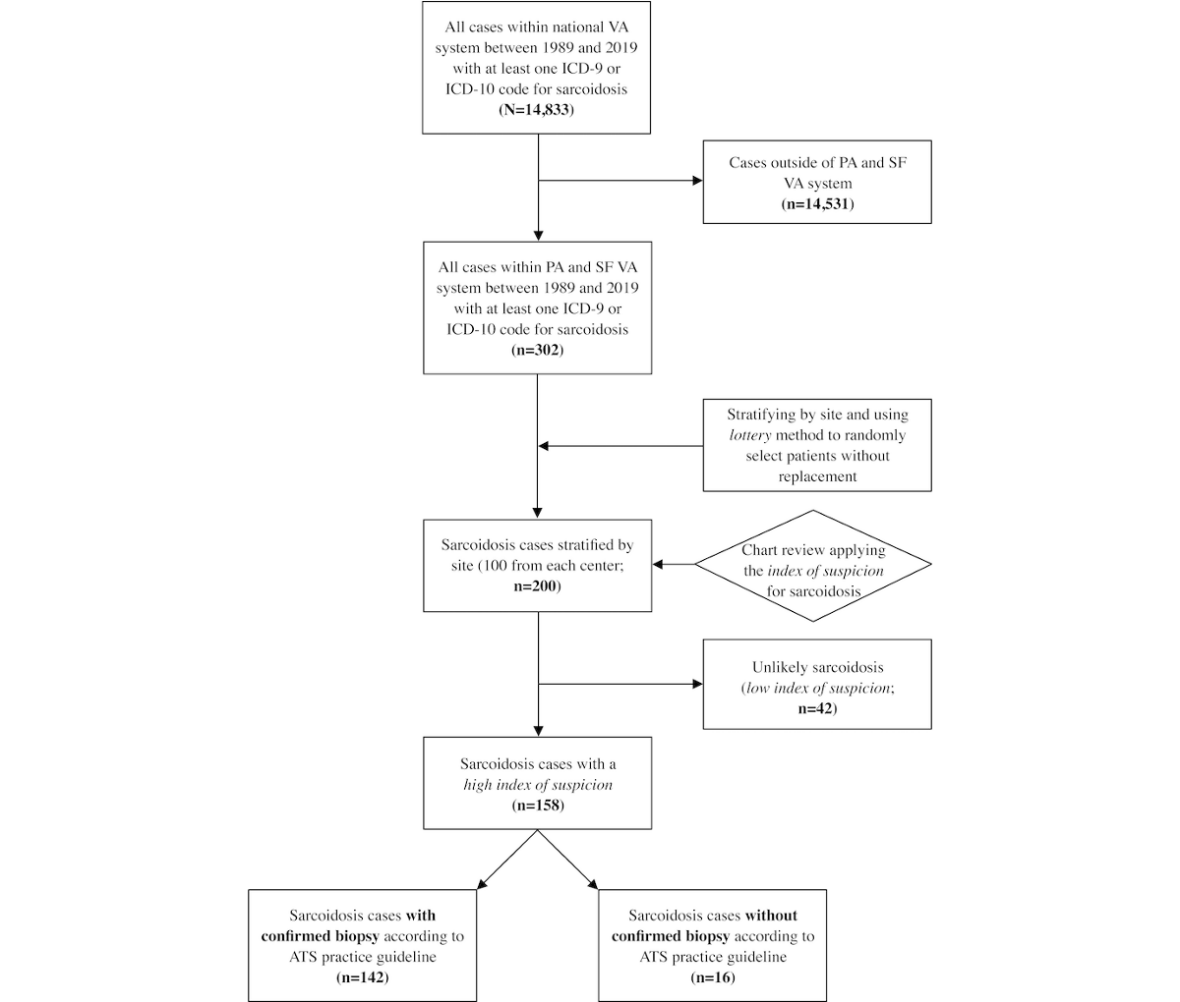
Strengthening the Reporting of Observational Studies in Epidemiology flowchart. Selection criteria for sarcoidosis cases. ATS: American Thoracic Society; ICD: International Classification of Diseases; PA: Palo Alto; SF: San Francisco; VA: Veterans Affairs.

To determine the statistical performance of using diagnostic codes (ICD-9 and ICD-10) in identifying patients with sarcoidosis from the EMR, initially, we identified patients with at least one claim (inpatient or outpatient) of ICD diagnosis code for sarcoidosis. To ascertain the true diagnosis of sarcoidosis based on the ATS diagnostic criteria (clinical, radiographic, and pathological findings, as well as exclusion of other causes) [3], 2 clinicians (MIS and IM) performed a comprehensive chart review. Of the 14,833 identified cases, a total of 200 (1.35%) were reviewed to limit the required chart review to a manageable level. As our access to the detailed medical records was limited to the two medical centers of San Francisco VA (SFVA) and Palo Alto VA (PAVA), the reviewed charts were selected from these two centers. We stratified the list of sarcoidosis cases from the 2 centers by site and used the *lottery* method to randomly select 100 patients from each site without a replacement [28] (Figure 1).

On the basis of the ATS practice guidelines, the diagnosis can be confirmed for those who had a biopsy consistent with sarcoidosis, as well as consistent clinical and radiological findings and no evidence for an alternative diagnosis. However, the ATS practice guideline committee acknowledged that there were clinical situations in which a confirmatory biopsy may not be indicated or possible. Accordingly, based on the ATS practice guideline, those patients without biopsies can be classified as probable sarcoidosis [3]. Therefore, given that not all suspected patients have a tissue biopsy in clinical practice, we generated an *index of suspicion* for sarcoidosis to identify patients with sarcoidosis (confirmed and probable) based on clinical and radiographic information, regardless of the availability of biopsy data, and to assess whether this approach would improve the diagnostic accuracy. The *index of suspicion* was applied to the initial cohort of patients with ICD codes for sarcoidosis (n=200). The clinical and radiological features were extracted from the available structured and unstructured data without including the histopathology results. If the patients were documented to have one or more of these features, they were assigned to the *high index of suspicion* group (Textbox 1); otherwise, the patients were assigned to the *low index of suspicion* group.

**Criteria to determine *high index of suspicion*.**

**Clinical and radiological features supportive of the diagnosis of sarcoidosis that were used for the determination of a *high index of suspicion*. Any patients with at least one of these features were included in the *high index of suspicion* group:**

- Clinical
  - Lofgren syndrome (defined as erythema nodosum, bilateral hilar lymphadenopathy, and polyarthralgia or polyarthritis)
  - Heerfordt syndrome (defined as facial nerve palsy, parotid gland enlargement, anterior uveitis, and low-grade fever)
  - Lupus pernio or erythema nodosum
  - Maculopapular or erythematous skin lesions or nodules
  - Facial nerve palsy
  - Symmetrical parotid enlargement
  - Optic neuritis, scleritis, uveitis, or retinitis
  - Lacrimal gland swelling
  - Evidence of granulomatous disease on direct laryngoscopy
  - Hepatomegaly or splenomegaly
  - Shortness of breath, dyspnea on exertion, cough, dizziness, or chest pain
  - Pulmonary function test with obstruction, restriction, or low diffusing capacity of the lungs for carbon monoxide
  - Cardiomyopathy, cardiac arrhythmia, or atrioventricular node block
  - Hypercalcemia, hypercalciuria, nephrolithiasis, or abnormal vitamin D levels
  - Elevated angiotensin-converting enzyme inhibitors or soluble interleukin-2 receptors
  - Bronchoalveolar lavage lymphocytosis
- Radiological
  - Bilateral hilar lymphadenopathy (chest radiograph, computed tomography, and positron emission tomography)
  - Computed tomography chest with perilymphatic nodules tracking the peribronchovascular bundle
  - Diffuse infiltrates (chest radiograph, computed tomography, and positron emission tomography) or computed tomography chest or chest radiograph with fibrosis
  - Cardiac magnetic resonance imaging or positron emission tomography–computed tomography consistent with sarcoidosis
  - Enlargement or nodules in liver or spleen (computed tomography, positron emission tomography, or magnetic resonance imaging)
  - Magnetic resonance imaging brain with increased inflammation
  - Extrathoracic enlarged lymph nodes (computed tomography, magnetic resonance imaging, and positron emission tomography)

We then further classified the patients into 3 groups. Patients with a high index of suspicion and documented histopathological evidence of nonnecrotizing granulomas were categorized into the group of *sarcoidosis with confirmed biopsy*. Patients with a high index of suspicion and either no documented biopsy in the EMR or a biopsy showing no histopathological evidence of nonnecrotizing granulomas were categorized into the group of *sarcoidosis without confirmed biopsy* (probable sarcoidosis). Finally, those with a low index of suspicion were categorized into the group of *unlikely sarcoidosis* (Figure 1).

Using the *index of suspicion* restricts the initially developed sarcoidosis cohort to capture those with a *high index of suspicion* for sarcoidosis from whom we identified confirmed cases. As we started with a random sample of those with sarcoidosis diagnostic codes, the further restriction of the sample to those with a *high index of suspicion* was still a random sample of the combination of both ICD codes and the *index of suspicion*. We compared the statistical performance of the two methods (ICD code alone vs ICD code with *index of suspicion*) to determine whether the use of this *index of suspicion* could improve the PPV of identification of sarcoidosis cases in the EMR.

This approach provides more information than just relying on ICD codes alone to develop robust computational sarcoidosis-specific algorithms consistent with the recent ATS practice guideline recommendations.

### Disease-Related Variables

Organ involvement was assessed based on the clinical history obtained from physicians’ notes and imaging and biopsy reports available in the computerized patient record system. For this assessment, to adjust for the variability in providers’ documentation, we adapted a set of criteria previously introduced in the National Institutes of Health–sponsored Genomic Research in Alpha-1 Antitrypsin Deficiency and Sarcoidosis (GRADS) study [29].

We collected the following data from the chart review: clinical site, gender, race, ICD-9 and ICD-10 codes for sarcoidosis (135 and D86, respectively), the pathological diagnosis from any available biopsy, organ involvement as described in Textbox 2, Scadding staging of chest x-ray (as described in radiology reports), history of bilateral hilar lymphadenopathy (based on radiology reports and clinical notes), pulmonary function test (PFT) pattern (as reported in PFT reports), the clinical status (acute, chronic, or remitting disease), and the treatment status of sarcoidosis.

Pathological diagnoses were categorized into *primary* histopathological if the data were available in the pathology report domains and *secondary* if the data were available only in the clinical note domains because of either a remote history of biopsy or because the biopsy had been performed outside the VA. The PFT reports at the SFVA and PAVA used Crapo reference equations to calculate the lower limit of normal values for spirometry and lung volume measurements.

Using the clinical data from chart abstraction, we classified the patients into the clinical phenotypes proposed by the GRADS study, with the exception of *multi-organ phenotype*, which we defined as the involvement of ≥3 organs.

**Organ involvement assessment for sarcoidosis (with and without confirmed biopsy).**

**Organ and assessment**

**Lung**

- Positive lung biopsy and positive mediastinal or hilar lymph node biopsy
- Chest x-ray, computed tomography (CT) chest, or positron emission tomography (PET) demonstrating bilateral hilar lymphadenopathy; CT chest with perilymphatic nodules tracking the peribronchovascular bundle; chest X-ray, CT chest, or PET with diffuse infiltrates; and CT chest or chest x-ray (CXR) with fibrosis
- Pulmonary function test (PFT) with obstruction, restriction, or low diffusing capacity of the lungs for carbon monoxide (DLCO)

**Skin**

- Positive skin biopsy
- Lupus pernio and erythema nodosum

**Eye**

- Positive conjunctival or scleral biopsy
- Optic neuritis, scleritis, uveitis, or retinitis

**Cardiac**

- Positive heart or pericardium biopsy
- Atrioventricular node block (second or third degree)
- Cardiomyopathy responsive to treatment
- Cardiac arrhythmia (eg, ventricular tachycardia)
- Cardiac magnetic resonance imaging (MRI) or PET-CT consistent with sarcoidosis

**Liver or spleen**

- Positive liver or spleen biopsy
- Enlargement or nodules in liver or spleen (CT, PET, or MRI)
- Abnormal liver enzymes

**Neurosarcoidosis**

- Positive brain or dura or peripheral nerve biopsy
- Clinical syndrome or symptoms consistent with central nervous system sarcoidosis along with a positive MRI

**Ear, nose, and throat**

- Positive biopsy from ear, nose, or throat
- Direct laryngoscopy consistent with granulomatous disease

**Multi-organ involvement**

- ≥3 organs involved based on other criteria in this table

### Statistical Analyses

All statistical analyses were performed with R software (The R Foundation for Statistical Computing) using RStudio (version 1.2.5). Descriptive statistics were computed to summarize the data. Categorical variables were presented as the frequency in percentages, and continuous data were presented as means and SDs. We estimated the PPV of the two aforementioned computational diagnostic criteria for sarcoidosis (ICD codes alone and ICD codes along with *index of suspicion*). We did not report the positive likelihood ratio, given that the specificity for using ICD codes alone could not be calculated because our study design did not include a review of noncases. The PPV for the criterion of using only the ICD code was calculated as the number of patients with an ICD code for sarcoidosis divided by the total number of patients verified to have sarcoidosis by chart review (*gold standard*). The PPV for the criterion of using the ICD codes and *index of suspicion* was calculated as the total number of patients with a high index of suspicion divided by the number of patients verified to have sarcoidosis by chart review (*gold standard*). The sensitivity of histopathology reports alone compared with chart review was calculated as the total number of patients with a high index of suspicion and confirmed biopsy divided by the number of patients verified to have sarcoidosis by chart review (*gold standard*). We computed 95% CIs using the exact binomial method. For our estimates, significance was defined as *P<*.05.

## Results

### Patients’ Characteristics

A total of 14,833 patients with at least one ICD-9 or ICD-10 diagnostic code of sarcoidosis were identified. The study cohort included patients identified by the ICD codes of sarcoidosis (n=200). Of the 200 patients, 158 (79%) had a *high index of suspicion* for sarcoidosis based on clinical or radiographic findings. Of these 158 patients, 108 (68.4%) were identified with the ICD-9 code of 135 and 50 (31.6%) with the ICD-10 code of D86, and 142 (89.9%) had confirmed sarcoidosis based on histopathological evidence of nonnecrotizing granuloma and were classified as having *sarcoidosis with confirmed biopsy*; the remaining 16 (10.1%) patients with a *high index of suspicion* did not undergo a biopsy and were classified as having *sarcoidosis without confirmed biopsy* (probable sarcoidosis; Figure 1). No patient had nondiagnostic biopsy results for sarcoidosis.

Table 1 summarizes the demographic data and baseline characteristics of patients with sarcoidosis (with and without confirmed biopsy). Among these patients, 89.9% (142/158) were men and there was a higher representation of African American patients than non-Hispanic White patients (85/158, 53.8%, vs 52/158, 32.9%, respectively). Overall, 90.5% (143/158) had a predominant pulmonary phenotype. Among these, 129 had PFT (36, 27.9%, 28, 21.7%, and 25, 19.4%, with restrictive, obstructive, and mixed patterns, respectively) and most were in Scadding stage II (47/143, 32.9%), followed by stage 0 and stage I (27/143, 18.9%, and 26/143, 18.2%, respectively). There was no significant difference in age between those who had a biopsy performed to diagnose sarcoidosis and those who did not (mean 65.5, SD 10.8, years vs mean 69.3, SD 10.3, years, respectively; *P*=.18). In terms of clinical phenotypes, 37.9% (60/158) had a *multi-organ* disease (≥3 organs; there were none with involvement of ≥5 organs), followed by stage II or stage III treated (45/158, 28.5%). Our study cohort did not include any individuals with acute presentation (acute, untreated). Some patients overlapped with multiple clinical groups.

**Table 1:** Distribution of characteristics and clinical phenotype groups of patients with sarcoidosis (with and without confirmed biopsy; N=158).

Characteristics				Sarcoidosis with confirmed biopsy (n=142), n (%)						Sarcoidosis without confirmed biopsy (n=16)^a^, n (%)						*P* value	
Age (years), mean (SD)				65.5 (10.8)						69.3 (10.3)						.18	
**Sex**																	.59^b^
		Male						127 (89.4)						15 (93.7)			
		Female						15 (10.6)						1 (6.3)			
**Race**																	.62^c^
		African American					74 (52.1)						11 (68.8)				
		Non-Hispanic White					49 (34.5)						3 (18.8)				
		Hispanic White					3 (2.1)						0 (0)				
		Unknown					12 (8.5)						2 (12.5)				
		Other					4 (2.8)						0 (0)				
**International Classification of Diseases codes for sarcoidosis**																	.60^b^
		International Classification of Diseases, Ninth Revision					98 (69)						10 (62.5)				
		International Classification of Diseases, Tenth Revision					44 (30.9)						6 (37.5)				
**Organ involvement**																	.38^c^
		Lung					86 (60.6)						12 (75)				
		Multi-organ (pulmonary without cardiac)					39 (27.5)						2 (12.5)				
		Multi-organ (pulmonary and cardiac)					4 (2.8)						0 (0)				
		Multi-organ (cardiac without pulmonary)					2 (1.4)						0 (0)				
		Multi-organ (neither cardiac nor pulmonary)					11 (7.7)						2 (12.5)				
**Pulmonary function test pattern^d^**																	.03^c^
	Obstructive				27 (19)						1 (6.3)						
	Restrictive				30 (21.1)						6 (37.5)						
	Mixed				20 (11.9)						5 (31.3)						
	Normal				39 (27.5)						1 (6.3)						
	Missing				26 (18.3)						3 (18.8)						
**Scadding stage^e^**																	.06^c^
	Stage 0					22 (15.5)						5 (31.3)					
	Stage I					23 (16.2)						3 (18.8)					
	Stage II					45 (31.7)						2 (12.5)					
	Stage III					17 (11.9)						4 (25)					
	Stage IV					22 (15.5)						0 (0)					
	Missing					13 (9.2)						2 (12.5)					
**Clinical phenotype group^f^**																	.06^c^
	Group 1: multi-organ		56 (39.4)						4 (25)								
	Group 2: nonacute, stage I, untreated		6 (4.2)						2 (12.5)								
	Group 3: stages II-III, treated		42 (29.6)						3 (18.8)								
	Group 4: stages II-III, untreated		14 (9.9)						2 (12.5)								
	Group 5: stage IV, treated		17 (11.9)						0 (0)								
	Group 6: stage IV, untreated		4 (2.8)						2 (12.5)								
	Group 7: acute sarcoidosis, untreated		0 (0)						0 (0)								
	Group 8: remitting, untreated		30 (21.1)						5 (31.3)								
	Group 9: cardiac sarcoidosis, treated		6 (4.2)						0 (0)								

^a^Probable sarcoidosis: cases with clinical and radiological features consistent with sarcoidosis and do not have confirmatory biopsies.

^b^Chi-square test.

^c^Fisher exact test.

^d^Evaluated based on pulmonary function test reports available in the computerized patient record system.

^e^Scored based on reviewers’ interpretation of imaging reports using Scadding staging. Stage 0: normal chest radiograph; stage I: hilar or mediastinal nodal enlargement only; stage II: nodal enlargement and parenchymal disease; stage III: parenchymal disease only; stage IV: end-stage lung disease (pulmonary fibrosis).

^f^Clinical phenotype groups [29]: some patients overlapped with multiple clinical groups. Group 1: multi-organ involvement, patients with ≥3 organs involved; group 2: nonacute, stage I, untreated: patients with nonacute sarcoidosis, stage I, never treated for sarcoidosis; group 3: stage II-III, treated: patients with nonacute sarcoidosis, stage II or III, formerly treated for sarcoidosis or treated within 3 months of data review; group 4: stage II-III, untreated: patients with nonacute sarcoidosis, stage II or III, never treated for sarcoidosis; group 5: stage IV, treated: patients with nonacute sarcoidosis, stage IV, formerly treated for sarcoidosis or treated within 3 months of data review; group 6: stage IV, untreated: patients with nonacute sarcoidosis, stage IV, never treated for sarcoidosis; group 7: acute sarcoidosis, untreated: patients with acute sarcoidosis (Lofgren syndrome); group 8: remitting, untreated: patients who have had no evidence of active clinical disease for >1 year; group 9: cardiac sarcoidosis, treated: patients with cardiac manifestations of sarcoidosis, formerly treated for sarcoidosis or treated within 3 months of data review.

### Diagnostic Accuracy of ICD Codes

We then calculated the PPV using ICD codes to identify VA patients who met the ATS definition of sarcoidosis from the VINCI database. For this calculation, we used the curated data set of 200 patients. The PPV of using only ICD codes was 79% (95% CI 78.6%-80.5%) for identifying sarcoidosis cases and 71% (95% CI 64.7%-77.3%) for identifying histopathologically confirmed sarcoidosis in the EMR. After chart review, the inclusion of the generated *high index of suspicion* to identify confirmed sarcoidosis cases increased the PPV significantly to 100% (95% CI 96.5%-100%) with 90% sensitivity of histopathology reports alone compared with chart review (Table 2).

**Table 2:** Contingency 2×2 table of using histopathology reports compared with high index of suspicion for sarcoidosis cases identification (N=200).

Among patients with International Classification of Diseases code for sarcoidosis			High index of suspicion^a^ (chart review)			
			Yes		No	Total
**Histopathology report^b^**						
	Confirmed sarcoidosis	142^c^		0^d^		142
	Not available^e^	16^f^		42^g^		58
	Total	158		42		200

^a^High index of suspicion for sarcoidosis based on both clinical and radiographic evidence but not biopsy.

^b^Available biopsies with primary or secondary histopathological reports.

^c^Sarcoidosis group with histopathological evidence of nonnecrotizing granuloma.

^d^No sarcoidosis group because of lack of sufficient clinical and radiological features consistent with sarcoidosis even in the presence of the histopathological evidence of nonnecrotizing granuloma.

^e^No biopsies were ordered or available in the electronic medical record.

^f^Probable sarcoidosis group without histopathological evidence of nonnecrotizing granuloma.

^g^No sarcoidosis group because of lack of sufficient clinical and radiological features consistent with sarcoidosis, in addition to the absence of the histopathological evidence of nonnecrotizing granuloma.

## Discussion

### Principal Findings

In this observational retrospective study of VA EMRs, we reviewed the medical records of 200 randomly selected patients with ICD diagnostic codes for sarcoidosis from the SFVA and PAVA medical centers (Figure 1). In this sample, we found that ICD diagnostic codes performed reasonably well with a PPV of 79% for detecting patients with sarcoidosis and 71% for detecting patients with histopathologically confirmed cases as defined by the ATS clinical practice guideline. After applying the developed *index of suspicion* to the initial cohort, we also demonstrated that including a *high index of suspicion* that incorporated information from radiology and clinical domains, but not histopathology, significantly increased the diagnostic accuracy to 100% (95% CI 96.5%-100%). The results of this study will help researchers and health care systems better understand the accuracy of using diagnostic codes alone versus using ICD codes with a *high index of suspicion* for sarcoidosis as classifiers in detecting a complex disease such as sarcoidosis in the EMR. Furthermore, the study highlighted other computational challenges in querying sarcoidosis cases and accurately extracting high-quality sarcoidosis-related research variables from the EMR. This approach could be adapted to develop automated chart review algorithms using additional data elements from structured and unstructured domains by applying advanced computational methodologies such as natural language processing (NLP) and machine learning.

The randomly selected cohort of veterans in this study with sarcoidosis (with and without confirmed biopsy) consisted of 89.9% (142/158) of men and 10.1% (16/158) of women. Although the gender distribution in our study was different from that in A Case Control Etiologic Study of Sarcoidosis [30], it is closely reflective of the demographics in the veterans’ population [31]. This study confirmed the higher prevalence of sarcoidosis in African American individuals (85/158, 53.8%) compared with non-Hispanic White individuals (52/158, 32.9%), a finding that many other epidemiological studies on sarcoidosis have previously reported [32-36]. At the same time, the study population was racially diverse, highlighting the potential utility of the VA EMRs for studying sarcoidosis in medically underserved populations [37]. In our study, the PPV was reasonable compared with the study conducted by Ungprasert et al [14] for detecting patients with sarcoidosis in the EMR. This difference could be due to not using the ICD-10 code and having a less diverse population (85% White vs 9% Black).

Using ICD codes alone to extract health information is far more convenient than the time-consuming process of manually reviewing narrative data sets in unstructured data. However, using ICD codes to identify sarcoidosis cases in large data sets with thousands of patients poses several practical challenges. First, given the heterogeneity of sarcoidosis, it is challenging to efficiently confirm the presence of the disease. The verification process requires careful analysis of the available narrative data such as progress notes, imaging reports, and pathology reports to establish the case definition based on the sarcoidosis diagnostic criteria [3]. Second, the precise identification of the type of organ involvement through the EMR is a complex process and requires a thorough review of unstructured data. Although there are subcodes for ICD diagnostic codes that aim to capture the involvement of various organs, health care providers may or may not be familiar with these subcodes and may or may not use them correctly.

Moreover, there are no specific ICD codes for classifying the involvement of some organs in sarcoidosis (such as the central nervous system or gastrointestinal tract) [38]. Third, ICD codes do not determine the extent of the disease, such as described by the stages of a chest x-ray [39], because of a lack of ICD codes for different stages of pulmonary sarcoidosis [38]. Analysis of pulmonary features requires a manual review of every patient’s radiology reports and cannot be performed using only ICD codes. Finally, ICD codes do not specify the various sarcoidosis presentations such as acute, remitting, or chronic disease [29,38]. Thus, they cannot be used to classify patients into the previously described phenotype groups.

The definition of clinical phenotypes has become an essential goal for the sarcoidosis scientific community because genetic studies have identified different patterns of gene expression associated with disease severity and disease course [40,41]. In 2015, the National Heart, Lung, and Blood Institute held a workshop to leverage current scientific knowledge and define platforms to address disease disparities, identify high-risk phenotypes, and improve sarcoidosis outcomes [25]. A total of 9 different steps and research strategies were recommended to expand the scope of sarcoidosis research, including EMR-based research, to provide a unified and multidisciplinary approach. Such an approach is expected to bring together stakeholders interested in reducing the burden and severity of sarcoidosis. However, the major barrier in the efficient use of EMR data is the accurate extraction of research-quality variables, case definitions, and outcomes [42]. Thus, the rapid identification of cases and extraction of relevant clinical variables from the EMR using computational phenotype algorithms have emerged as an important next step in EMR-based research. Furthermore, computational phenotype definitions are also essential for conducting pragmatic clinical trials and comparative effectiveness research, increasing the health care system’s capacity to effectively deliver precision medicine for patients with sarcoidosis [43].

The two most applied approaches to defining computational phenotypes are (1) a *high-throughput* phenotype algorithm using only structured data (traditionally, the ICD diagnosis codes) and (2) a *low-throughput* phenotype algorithm that accesses structured and unstructured data to develop a sequential flowchart that should end with a case definition. Such a low-throughput approach uses high-performance computational tools such as NLP to process text and extract information using linguistic rules, thereby eliminating the need for a labor-intensive manual review by researchers [7]. Accordingly, this approach is expected to streamline the development of registries and help enrich EMR-based research studies [44]. Our study highlights the need to develop such automated methods to improve the computational case definition of sarcoidosis. Besides, there are other high-quality sarcoidosis-related research variables, including determining the date of the diagnosis, organ involvements, Scadding stages, and the clinical status (acute, chronic, or remitting disease). This approach will assist in automating the extraction of pre-existing or novel clinical phenotypes more precisely and efficiently from the EMR.

### Limitations

Our study includes several limitations. First, primary histopathological reports were not available for all the patients. In the cases where the biopsy report was unavailable (either because of a remote history of the biopsy or because the biopsy had been performed outside the VA), we relied on the *secondary* histopathological reports documented in the providers’ narrative within the clinical notes. This approach made the diagnosis of sarcoidosis less robust because the confirmatory biopsy reports in these patients could not be directly verified. However, we used the *index of suspicion* approach to define probable sarcoidosis cases regardless of whether a confirmatory biopsy report was available, which is consistent with the diagnostic algorithm recommended by the ATS practice guideline [3]. Second, our definition of *multi-organ phenotype* involved ≥3 organs, instead of ≥5 organs as proposed by the GRADS study [29]. We chose this approach because none of the evaluated patients were documented to have involvement of ≥5 organs, thus avoiding having no patients with *multi-organ phenotype*. Lack of patients with involvement of ≥5 organs could be due to EMR-related limitations such as missing data and variability in documentation among providers or simply because these patients were cared for at non-VA tertiary medical centers. Third, the generalizability of our findings obtained from VA EMRs to other populations could be limited because the veterans form a special population with a different demographic distribution and exposure from the general population. However, the EMR data of the VA health care system cover >22 million veterans across the United States and >14,000 patients with sarcoidosis ICD diagnosis codes, providing an enormous number of patients to study a rare disease. Moreover, the number of patients whose records were examined in this study was 200, which could be considered a small sample size. However, we analyzed data from nearly two-third of all patients with diagnostic codes for sarcoidosis in the VA health care system across northern California.

### Conclusions

Although ICD codes can be used as reasonable classifiers to identify sarcoidosis cases within EMRs with a PPV of 79%, using computational algorithms to extract clinical and radiographic information (*index of suspicion*) from unstructured data could significantly improve the accuracy of case identification. Furthermore, to increase the efficiency of identifying sarcoidosis cases from large health care databases, more studies are required to develop a novel sarcoidosis-specific computational phenotype algorithm using automated emerging methods (such as machine learning and NLP). Moreover, our study sets the stage for promoting research on developing other such algorithms aiming to generate high-quality sarcoidosis-related research variables, such as determining the date of the diagnosis, organ involvements, Scadding stages, and the clinical status (acute, chronic, or remitting disease).

## Abbreviations

ATS: American Thoracic Society
EMR: electronic medical record
GRADS: Genomic Research in Alpha-1 Antitrypsin Deficiency and Sarcoidosis
ICD: International Classification of Diseases
NLP: natural language processing
PAVA: Palo Alto Veterans Affairs
PFT: pulmonary function test
PPV: positive predictive value
SFVA: San Francisco Veterans Affairs
VA: United States Department of Veterans Affairs
VINCI: VA Informatics and Computational Infrastructure
